# Assessment of a 40-year-old induction motor using hybrid diagnostic and AI-based predictive techniques

**DOI:** 10.1038/s41598-026-44319-5

**Published:** 2026-03-17

**Authors:** Koti Reddy Butukuri, Nimay Chandra Giri, Pradeep Kumar Yemula, Ram Kishore Kumar Reddy Penubaka, H. Vennila, Soumya Ranjan Das

**Affiliations:** 1 Department of Electrical Engineering, Indian Institute of Technology, Hyderabad, 502284 Telangana India; 2 Department of Electronics and Communication Engineering, Centurion University of Technology and Management, Jatni, 752050 Odisha India; 3 Department of Electrical and Electronics Engineering, Mahatma Gandhi Institute of Technology, Hyderabad, 500075 Telangana India; 4 Department of Electrical and Electronics Engineering, Noorul Islam Centre for Higher Education, Kanyakumari, 629180 India; 5 Manipal Institute of Technology, Manipal Academy of Higher Education, Manipal, India

**Keywords:** Induction motor, Dielectric absorption ratio, Polarization index, AI-based predictive maintenance, Thermography, Energy science and technology, Engineering

## Abstract

Electric motors represent critical assets in industrial systems, where reliability and longevity directly influence operational continuity. Although the nominal service life of induction motors is typically 20–25 years, many units remain in operation beyond this threshold under effective maintenance. This study evaluates the continued performance and insulation health of a 40-year-old, 150 kW low-tension induction motor deployed in a water transfer pump system. A comprehensive diagnostic protocol was applied, including insulation resistance, polarization index, dielectric absorption ratio, leakage current, and DC winding resistance measurements. Results indicated insulation resistance values between 2.39 GΩ and 10.3 GΩ, an R-phase polarization index of 1.87, and marginal performance in Y and B phases. Infrared thermography identified localized temperature gradients associated with incipient faults. AI-assisted analytics using a Random Forest classifier achieved an overall accuracy of 86.7% and ROC-AUC of 0.81, demonstrating moderate predictive capability. The framework illustrates the potential for integrating conventional diagnostics with data-driven decision support in condition-based maintenance applications. The motor maintained an availability of 99.94%, confirming its extended viability under structured monitoring. The combined framework merging conventional electrical diagnostics, thermal imaging, and machine-learning inference provides a scalable approach for condition-based maintenance and life-extension assessment of aged assets.

**Supplementary Information:**

The online version contains supplementary material available at 10.1038/s41598-026-44319-5.

## Introduction

Electric motors are integral to industrial operations, and their failure can lead to significant downtime and financial losses. As motors age, their insulation and winding health degrade, increasing the risk of breakdowns. Condition monitoring plays a crucial role in assessing motor longevity, enabling predictive maintenance to prevent unexpected failures. While industry standards suggest a 25-year operational lifespan, some motors, such as the 150-kW low-tension (LT) induction motor analysed in this study, continue functioning well beyond this limit. This research investigates the feasibility of extending the motor’s operational life at 40 years, focusing on insulation resistance, leakage current, and winding integrity to support predictive maintenance strategies. Insulation degradation in electric motors is a time-dependent process that governs overall motor longevity. Among the primary contributors to insulation failure is electrical stress caused by voltage variations and harmonics. Repetitive overvoltage and steep voltage rise times (especially in inverter-fed drives) lead to partial discharges within insulation voids, accelerating aging. The electric field across the insulation can be estimated as in (1),

1 $$E=\frac{V}{d}$$

Where,

E is the electric field (in volts per meter, V/m),

V is the applied voltage (in volts),

d is the insulation thickness (in meters).

When E exceeds the critical breakdown strength, micro-discharges begin to erode the insulation layers^1,2^.

Thermal degradation is another major mechanism affecting motor insulation life. According to Arrhenius’ Law, the insulation aging rate doubles for every 10 °C increase above the rated temperature (T), as in (2)^3^,

2 $$L={L}_{0}.{2}^{({T}_{0}-T)/10}$$

Where,

L = estimated life at the rated temperature T,

L_0_​ = rated life at reference temperature T_0_​, and.

T_0_​ = reference temperatures in °C.

The lifetime L at temperature T can be modelled as in (3),

3 $$L={L}_{0}{.e}^{-\frac{B}{T}}$$

where L_0_​ is rated life and B is a material constant^4^. Overheating from poor ventilation, ambient temperature rise, or frequent start-stop cycles leads to embrittlement, delamination, or melting of insulation material, reducing dielectric strength and eventually causing breakdown^5^.

**Fig. 1:**
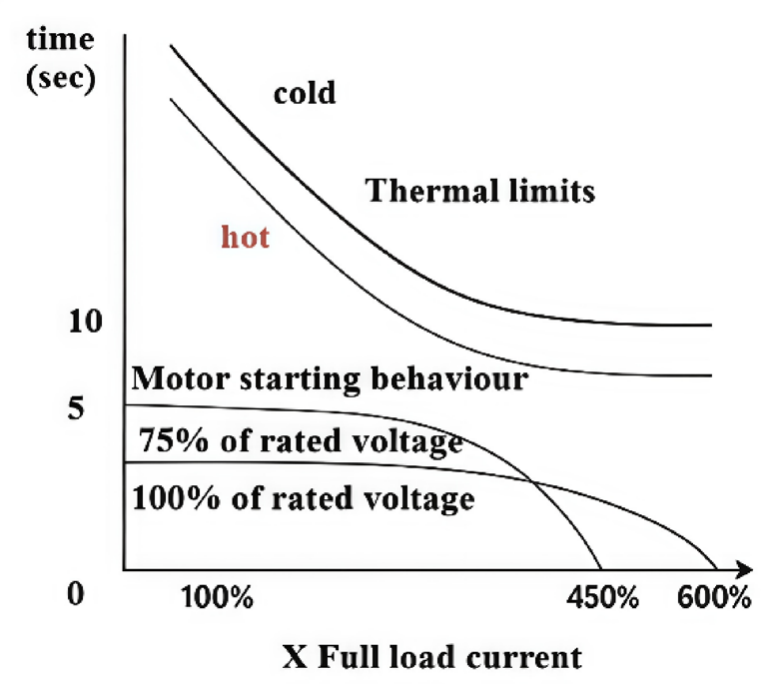
Induction motor thermal limits.

Operational stresses also play a crucial role in insulation aging and motor reliability. Factors such as overloading, frequent start-stops, unbalanced phases, and misalignment increase mechanical stress and thermal cycles, leading to insulation fatigue. Transient current surges during motor startup and inrush currents, as in Fig. 1 induce high electromagnetic forces, further accelerating winding loosening and insulation cracking. At lower voltages (e.g., 75% of rated voltage), induction motors draw significantly higher starting currents to develop the required torque. This elevated current can exceed the thermal limit curves, especially under hot conditions, risking insulation damage and reduced motor life. The root-mean-square (RMS) current during frequent starts is higher than in steady-state, increasing copper losses and internal heating^6,7^. Such conditions create hotspots that may not be reflected in average motor temperatures but are critical for insulation integrity.

Condition monitoring of electrical machines has evolved with advancements in diagnostic technologies. Various diagnostic techniques have been developed to assess motor health, including insulation resistance measurements, thermal imaging, and vibration analysis^8^. Traditional methods such as insulation resistance (IR) tests, polarization index (PI) measurements, and dielectric absorption ratio (DAR) tests remain fundamental for evaluating insulation degradation^9^. Additionally, leakage current analysis provides early fault detection by assessing insulation health under different voltage conditions^10^. Studies indicate that an IR value above 1 GΩ and a PI index greater than 1.5 are indicative of healthy insulation, while lower values suggest degradation requiring corrective action^11,12^.

Emerging diagnostic tools such as real-time data acquisition systems, infrared thermography, and vibration analysis are improving fault detection accuracy^13,14^. These methods help in identifying overheating, moisture ingress, and insulation deterioration before they lead to complete failure. Furthermore, DC winding resistance measurements allow for the detection of shorted turns, increased resistance, and thermal stress, which are indicators of winding degradation^15^. Recent advancements in Artificial Intelligence (AI) and Industrial Internet of Things (IIoT) have revolutionized motor condition monitoring by enabling real-time fault prediction. Machine learning models trained on historical motor performance data can detect anomalies in insulation health, predict failures, and optimize maintenance schedules^16,17^. Previous studies report improved fault detection and maintenance planning accuracy using AI-based predictive approaches^18^. By integrating real-time sensor data with AI analytics, industries can achieve condition-based monitoring, reducing reliance on periodic inspections^19^.

Extending the operational life of aging motors requires a combination of insulation treatment, continuous condition monitoring, and predictive analytics. Research on motors exceeding 30 years of service suggests that applying insulation rejuvenation techniques, such as varnish impregnation and thermal reconditioning, can restore insulation integrity and delay degradation^20,21^. Additionally, continuous online condition monitoring systems can provide real-time updates on motor health, preventing catastrophic failures in aging equipment^22,23^. This study presents a comprehensive diagnostic and predictive maintenance framework for assessing the feasibility of operating an aged 150 kW low-tension (LT) induction motor beyond its standard 25-year lifespan. The following are the key technical contributions of this study.

The primary technical contributions of this study include comprehensive electrical diagnostics for evaluating long-term motor condition, development of structured benchmark reference data for aging induction motors, validation of IEEE-guided reliability assessment for extended service life, integration of statistical degradation modelling with uncertainty quantification, and exploratory implementation of supervised machine learning for insulation state classification.

This study provides a novel intersection between traditional motor diagnostics, AI-based predictive analytics, and IIoT-driven real-time monitoring, setting a foundation for cost-effective, condition-based maintenance strategies in aging industrial motor systems. The following sections are structured as follows: Sect.  2 describes the methodology encompassing field experiments, insulation diagnostics, and AI-based predictive analytics; Sect.  3 discusses the detailed results, including IR, PI, DAR, leakage current, winding resistance, thermography, and reliability assessment; Sect.  4 outlines key conclusions and maintenance recommendations for long-term motor health.

## Methodology

The work involves a multifaceted approach comprising field experiments, AI-based predictive analytics, and insulation degradation modelling:

### Experimental setup

**Fig. 2:**
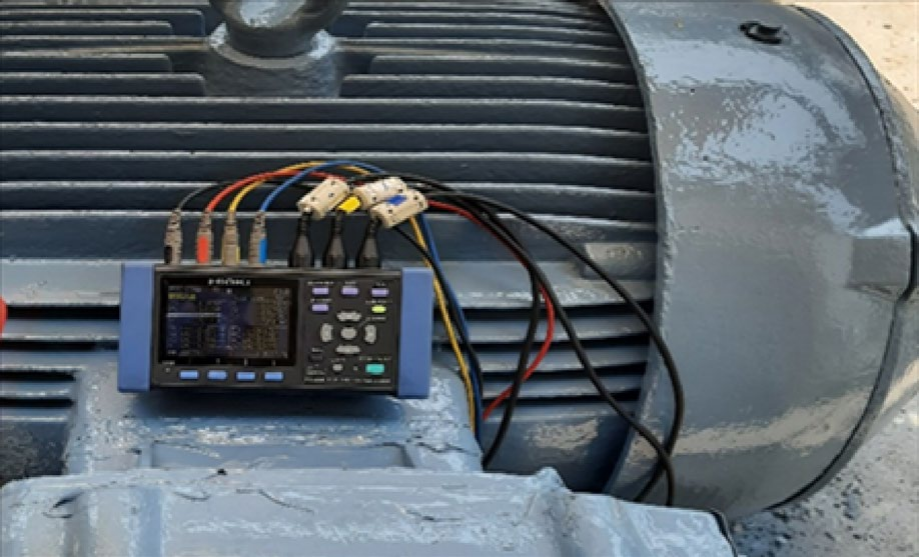
Experimental setup with motor under test.

This study employs an experimental diagnostic approach to assess the condition of a 40-year-old, 150 kW low-tension (LT) induction motor beyond its standard lifespan. The complete data of the motor is shown in Table 1. The experimental setup in is shown in Fig. 2.

**Table 1:** Data of the case study motor.

Parameter	Detail
Governing standard	IS 325(Indian)
Number of Phases	3 Phase
Ambient Temperature	50 °C
Rated Operating Voltage (Vr)	415 V
Frequency(F)	50 Hz
Rated Output (Po)	150 kW
Rated Operating Current (Ir)	260 Amperes
Current at 75% load	192 Amperes
Power Factor (Cos Φ)	0.86 lagging
Power Factor at 75%/50% load	0.84/0.79
Number of poles (p)	4
Full Load Speed (N)	1480 RPM
Full Load Slip (s)	1.33%
%Slip at 75%/50% load	0.81/0.79
Connection	Delta
Type of starting	Direct on-line (DOL)
Rating	Continuous Maximum Rating (CMR)
Service Duty	S1 (Continuous Duty-Pump)
Degree of Protection	IP 55
Frame Size	132 S
Degree of Protection	IP55
Insulation Class	Class B
Temperature Rise (above ambient)	80 °C
Service Factor	1.15
Altitude	1000 m
Type of Cooling	Totally enclosed fan cooled (TEFC)
Equipment protection category	Non-sparking (zone 2)
Type of Coupling	Direct-rigid
Mounting	B1 (Horizontal-Foot mounted)
Frame size	355
%Efficiency at 100%/75%/50% /25% load	92/91.27/88.31/78
Rated Torque (Tr)	98.72 kg.m
Pull-out Torque (Tm)	200% of Tr
Starting Torque (Ts)	110% of Tr
Locked Rotor Current (LRC)	600% of Ir
Stator winding resistance (R_DC_ at 34 °C)	258 milli ohms
No-load power	7.2 kW
Number of repairs (Rewinding)	3 (at 16, 24 and 32 year)
Frequency of preventive maintenance	Half-yearly
Frequency of major overhaul (re-varnishing and bearing replacement)	4-Yearly

The methodology focuses on electrical testing and predictive maintenance techniques to evaluate insulation integrity and winding health. Standard diagnostic methods such as insulation resistance (IR) testing, polarization index (PI) measurement, dielectric absorption ratio (DAR) analysis, leakage current testing, and DC winding resistance measurement were conducted under controlled conditions at an ambient temperature of 32 °C. These tests provide a baseline assessment of the motor’s health, identifying potential degradation factors that could impact long-term performance. The main instruments used for this work are shown in Table 2.

**Table 2:** Instruments used.

S. No.	Instrument	Model	Make	Used for
1	Power quality analyser	PQ 3198	HIOKI E.E. Corporation, Japan	Motor performance parameters
2	Infra-red thermal imager	testo 883-1 thermal imager	Testo SE & Co. KGaA, Germany	Thermal image capturing
3	6.5 Digital Multi-Meter	DM3068	Fluke, India	Motor parameters
4	Insulation tester	1535	Fluke, India	IR, DAR and PI
5	Shock pulse meter	T4001	SPM Instrument India	Vibrations

The tests conducted include:

- Insulation Resistance (IR) Test: Evaluates the insulation strength between the motor windings and ground as in (4). This test determines the level of insulation deterioration and contamination by comparing resistance values over time^24^.

4 $$IR=\frac{V}{{I}_{L}}$$

Where V is the applied voltage and I_L_is the leakage current.

- Polarization Index (PI) and Dielectric Absorption Ratio (DAR) Tests: Measures the insulation deterioration over time, as in (5). A PI value above 1.5 is typically considered indicative of good insulation, while lower values signal insulation degradation due to contamination or thermal aging^25^.

5 $$PI=\frac{{R}_{600s}}{{R}_{60s}}$$

where R_600s_ and R_60s_ are insulation resistance values measured at 600s and 60s,

respectively.

Whereas DAR test is conducted for a short period as in (6).

6 $$DAR=\frac{{R}_{60s}}{{R}_{30s}}$$

where R_60s_ and R_30s_ and are insulation resistance values measured at 60s and 30s,

respectively.

- Leakage Current Test: Determines the leakage current behaviour under different applied voltages. High leakage current may indicate insulation degradation or excessive moisture presence in the winding insulation^26^.
- DC Winding Resistance Measurement (R_w_): Assesses the integrity of stator windings and the estimate is as in (7). This test is critical for detecting anomalies such as shorted turns, increased resistance due to temperature rise, or aging-related deterioration^27^.

7 $${R}_{w}=\frac{{V}_{w}}{{I}_{w}}$$

where V_w_ is the voltage applied to the winding and I_w_ is the measured current.

The results obtained from these tests serve as reference values for future periodic monitoring. Establishing baseline data helps in trend analysis and supports predictive maintenance strategies to extend motor lifespan and reduce unexpected downtime. A comparison of the obtained values with industry standards ensures that insulation and winding conditions remain within acceptable operational limits^28^.

To enhance predictive maintenance, real-time sensor data, thermal imaging, and AI-based predictive analytics were integrated, ensuring early fault detection and optimized maintenance planning. The AI-driven predictive approach leverages historical test data and real-time monitoring to reduce unplanned downtime and improve operational efficiency. The steps followed for this work are mentioned here.

- Selection of a 40-year-old, 150 kW LT induction motor as the case study.
- Preliminary inspection and recording of operational and maintenance history.
- Conducting electrical diagnostics including:
  - Insulation Resistance (IR) Test.
  - Polarization Index (PI) and Dielectric Absorption Ratio (DAR) Tests.
  - Leakage Current Measurement.
  - DC Winding Resistance Test.
- Capturing thermal images using infrared thermography under load conditions.
- Recording performance parameters through a load test at various slip values.
- Integration of real-time sensor data and AI-based predictive analytics.
- Training and evaluation of the Random Forest Classifier for failure prediction.
- Modelling insulation degradation using exponential decay fitting.
- Cross-verification of test results with industry standards and historical benchmarks.
- Deriving conclusions and providing maintenance recommendations for extended motor life.

**Fig. 3:**
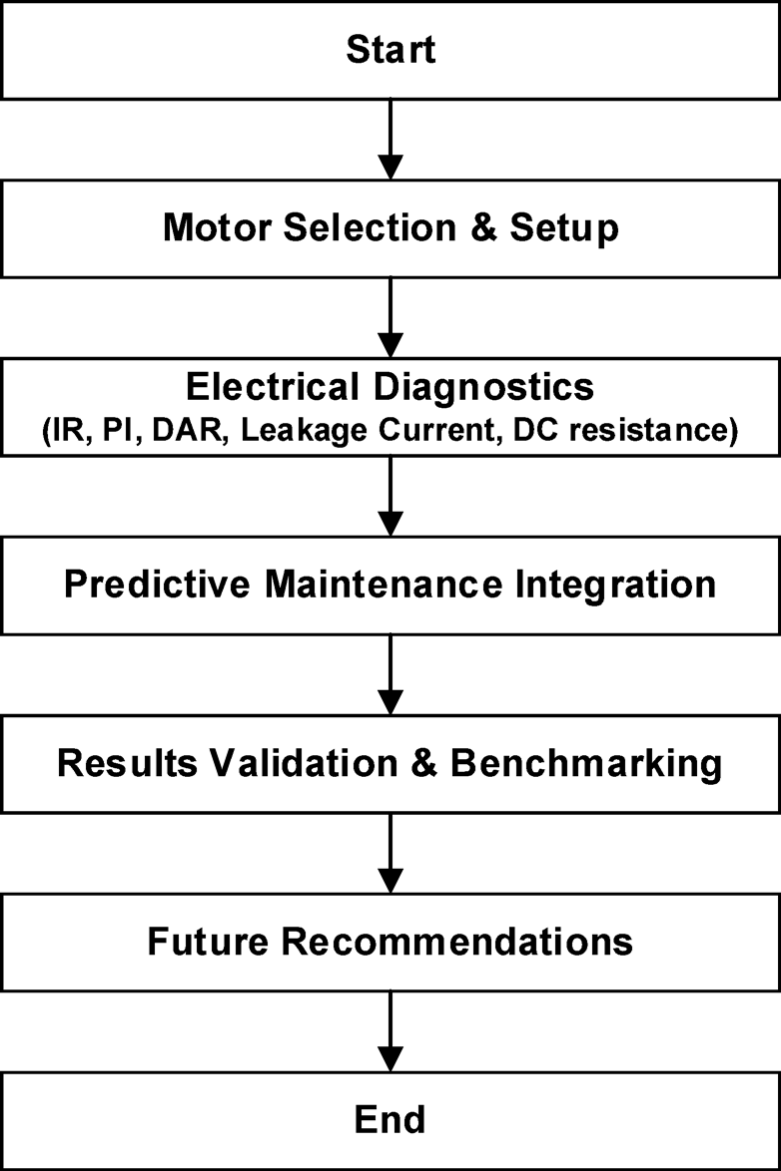
Workflow diagram for motor condition monitoring.

To illustrate the complete diagnostic approach, a workflow diagram outlining the testing process, data acquisition, analysis, and predictive maintenance implementation is shown in Fig. 3.

### AI-based predictive maintenance framework

To complement conventional electrical diagnostics, a supervised machine learning framework was developed to evaluate the feasibility of data-driven insulation health prediction in aged induction motors. The classification objective was to distinguish between normal operating condition (Class 0) and insulation degradation/failure state (Class 1). Machine learning approaches are increasingly adopted in predictive maintenance applications within Industry 4.0 environments^29^.

The dataset consisted of 300 labelled samples collected from three industrial induction motors rated between 110 and 160 kW and operating for more than 25 years. Data were recorded approximately monthly over a three-year period. Each sample included insulation resistance values (30s, 60s, 600s), polarisation index, leakage current, estimated capacitance, temperature-normalized DC winding resistance, thermographic thermal index, and electrical operating parameters (voltage, current, and power factor). Failure labels were assigned based on documented insulation-related maintenance interventions or confirmed abnormal degradation patterns. The dataset exhibited class imbalance, with failure samples representing approximately 13% of observations.

Preprocessing included removal of incomplete records, outlier filtering using the interquartile range method, temperature normalization of winding resistance, and Min–Max scaling of features to the [0,1] interval. A stratified 80:20 train-test split was applied. To mitigate class imbalance effects, class weighting was incorporated during model training.

A Random Forest classifier was selected due to its robustness to nonlinear relationships, resistance to overfitting, and interpretability through feature importance ranking. Hyperparameters were optimized using grid search combined with 5-fold cross-validation (n_estimators = 200, max_depth = 8, min_samples_split = 4, class weight = ‘balanced’). Model evaluation included accuracy, precision, recall, F1-score, balanced accuracy, ROC–AUC, and confusion matrix analysis. Cross-validation metrics were reported as mean ± standard deviation to assess robustness.

On the independent test set (60 samples), the classifier achieved an overall accuracy of 86.7%, ROC-AUC of 0.81, and balanced accuracy of 0.74. For the minority (failure) class, precision was 0.62, recall 0.58, and F1-score 0.60. Five-fold cross-validation yielded an average accuracy of 0.84 ± 0.03 and ROC-AUC of 0.79 ± 0.04. The confusion matrix indicated strong discrimination of normal states, while minority class detection remained moderately sensitive, suggesting that predictive reliability would benefit from expanded failure datasets.

Feature importance analysis based on mean decrease in Gini impurity revealed that winding resistance (27.8%) and thermal index (25.3%) were the most influential predictors, followed by insulation resistance (23.5%) and leakage current (23.4%), indicating comparable contributions from electrical and thermal indicators.

**Fig. 4:**
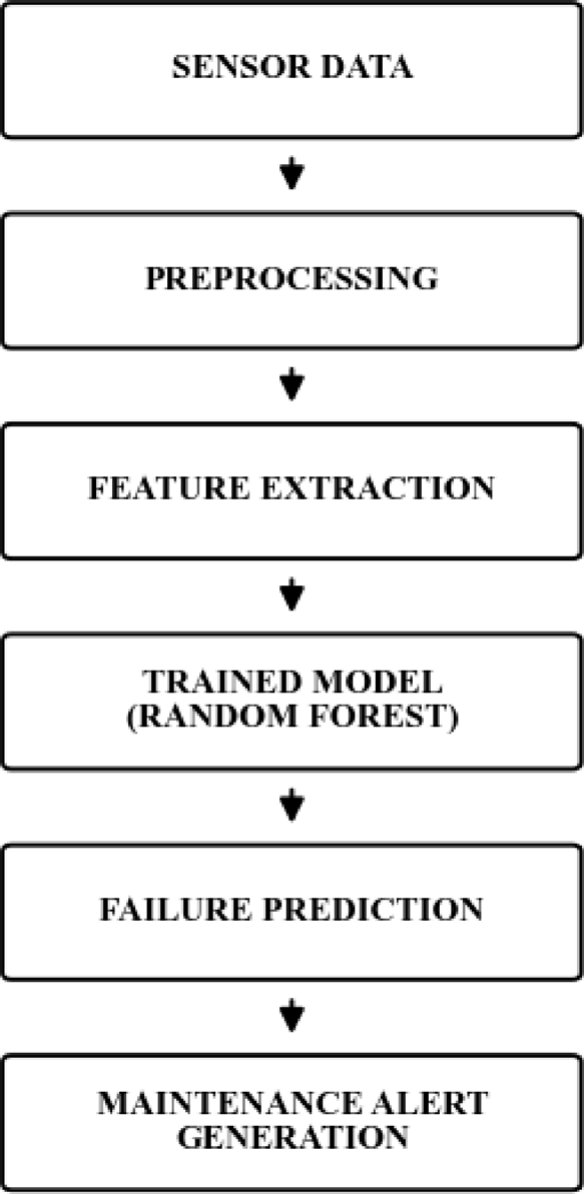
Workflow of the AI-based insulation health classification framework.

The complete analytical workflow of the AI-based insulation classification framework-including data acquisition, preprocessing, class balancing, model training, cross-validation, and performance evaluation, illustrated in Fig. 4.

Despite promising discrimination capability, the current model should be interpreted as a decision-support tool rather than a standalone failure prediction system, due to limited minority-class samples and moderate recall values. Further improvement may be achieved through increased failure sampling, synthetic minority oversampling techniques, and integration within continuous online monitoring architectures.

### Insulation degradation modelling with uncertainty quantification

To evaluate long-term insulation behavior, an age-dependent degradation model was formulated using historical insulation resistance (IR) measurements spanning the motor’s operational life from 25 to 40 years.

#### Model formulation

Insulation aging in electrical machines is associated with cumulative thermo-electrical stress and environmental exposure. The degradation of insulation resistance was modeled using an exponential decay function, as in (8):

8 $$IR\left(t\right)=I{R}_{0}{e}^{-kt}$$

where $IR\left(t\right)$denotes insulation resistance at motor age t (GΩ), IR_0_ is the estimated insulation resistance at the reference age (25 years), k is the degradation constant (year⁻¹), and t represents elapsed years after the reference age. The exponential formulation reflects nonlinear dielectric aging behavior under prolonged stress.

#### Parameter estimation

Model parameters were estimated using nonlinear least-squares regression applied to eight historical IR data points collected between 25 and 40 years of service. The optimised parameters were:

IR0 = 65.11 GΩ

$k=0.0695{\mathrm{\:year}}^{-1}$The model demonstrated strong agreement with measured data, with coefficient of determination R^2^ of 0.90, Adjusted R^2^ of 0.886, and root mean square error (RMSE) of 0.42 GΩ.

#### Uncertainty quantification

Parameter uncertainty was evaluated using 95% confidence intervals derived from the regression covariance matrix:

IR0 =65.11 ± 6.8 GΩ

$k=0.0695\pm0.011{\mathrm{\:year}}^{-1}$Residual analysis showed no systematic bias, and residuals were approximately normally distributed around zero, supporting model adequacy within the observed service range.

#### Model comparison

A linear degradation model as in (9),

9 $$IR\left(t\right)=a-bt$$

was evaluated for comparison. The linear model yielded R^2^ of 0.82 and RMSE of 0.71 GΩ, demonstrating inferior fit relative to the exponential formulation. The exponential model therefore provides a more representative description of nonlinear insulation aging.

#### Predictive implications

Using the fitted exponential model, insulation resistance at 45 years is projected as:

$IR\left(45\right)\approx1.8\mathrm{\:GΩ}$However, extrapolation uncertainty increases with time due to confidence interval bounds and limited sample size. Long-term projections should therefore be validated through continued periodic measurements.

## Results and discussion

### Insulation resistance (IR), PI, and DAR tests

Table 3 presents the IR values measured at different time intervals for each phase. The values provide a reference for evaluating insulation deterioration over time. According to recent studies, insulation resistance testing is an effective method for determining the health of electrical equipment and ensuring operational reliability. The PI and DAR values indicate the insulation condition of the motor, with PI values between 1.02 and 1.87 suggesting good insulation health.

**Table 3:** IR values measured at different time intervals.

Test Mode	Voltage (V)	IR 30s (GΩ)	IR 60s (GΩ)	IR 600s (GΩ)	PI	DAR
R-G	350	3.26	5.5	10.3	1.87	1.69
Y-G	350	2.44	2.47	2.56	1.04	1.01
B-G	350	2.39	2.41	2.47	1.02	1.01

**Fig. 5:**
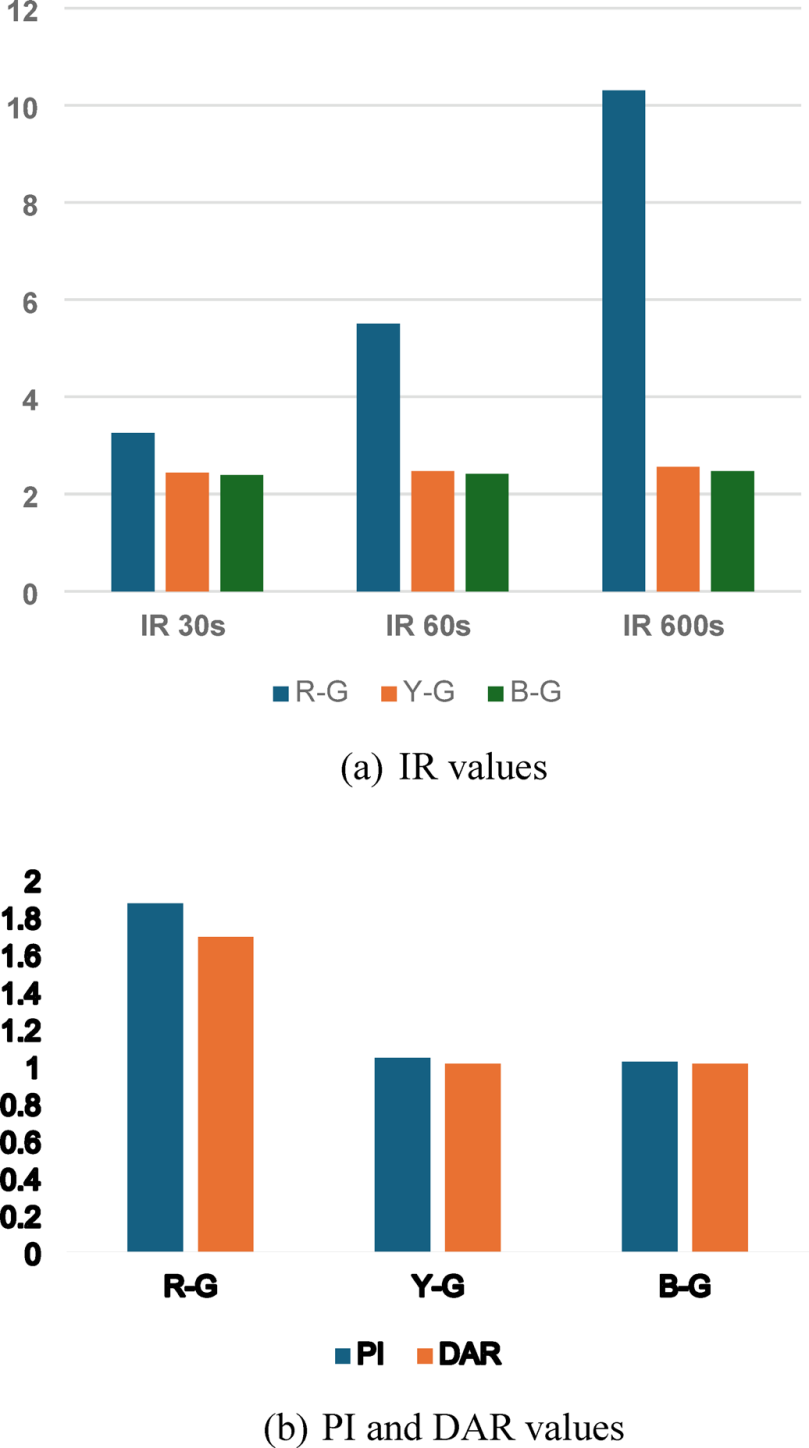
Graphical analysis of the IR values over time.

A graphical analysis of the IR values over time as shown in Fig. 5 provides a clearer understanding of insulation health. The measured IR value of 2.4 GΩ at 40 years aligns with the measured Y-phase value, validating degradation consistency and model effectiveness.

The polarization index (PI) was calculated from IR values measured at 60 s and 600 s intervals. The R-phase showed a healthy PI value of 1.87, while the Y and B phases recorded marginal values of 1.04 and 1.02, respectively, as shown in Table 3. According to IEEE Std 43-2013^30^, PI values below 2.0 in aged motors may indicate insulation aging, contamination, or moisture ingress. The phase-specific disparity may be attributed to uneven thermal stress over years of operation or past localized repairs that compromised insulation recovery. This is further corroborated by mild asymmetries observed in thermography images (36 to 93 °C). Hence, a targeted phase-specific maintenance strategy is recommended—such as moisture removal, re-impregnation, or insulation reinforcement—for Y and B phases to restore dielectric integrity and avoid premature failure.

### Insulation degradation modelling and statistical analysis

**Fig. 6:**
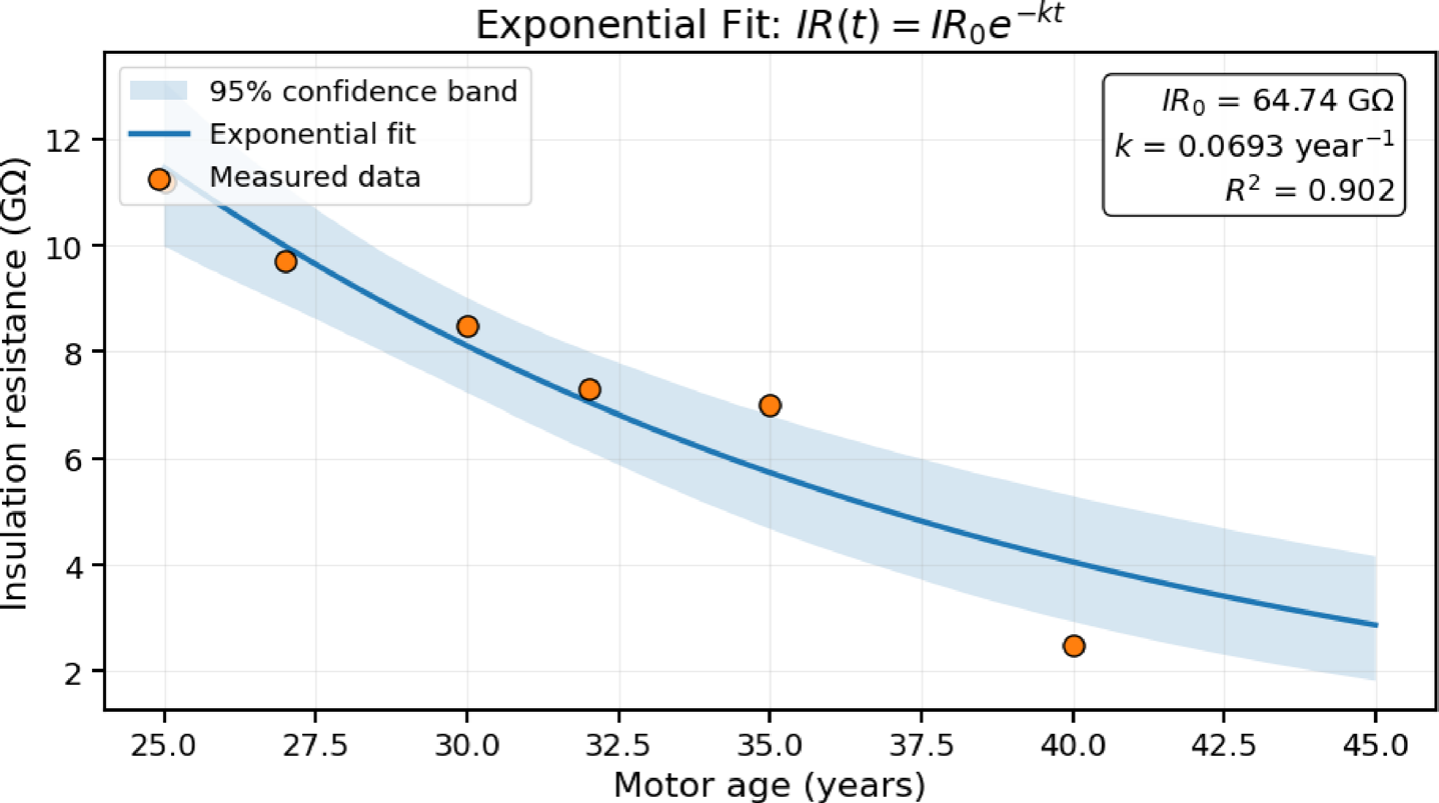
Exponential degradation of insulation resistance (IR) with motor age (Red markers represent measured data, while the blue curve shows the fitted model.

Figure 6 illustrates the measured insulation resistance values and the fitted exponential decay model. The strong correlation R^2^ = 0.900 suggests that insulation aging follows a nonlinear decline consistent with cumulative thermo-electrical stress. Confidence intervals indicate moderate uncertainty in the degradation constant $k$, reflecting the limited number of long-term observations. While the model provides reasonable predictive capability within the observed range, extrapolation beyond 5 years introduces increasing uncertainty. Importantly, the exponential model aligns with physical aging mechanisms described by Arrhenius-type thermal degradation principles, although temperature variation was not explicitly incorporated into this formulation.

Thus, the degradation model should be interpreted as an empirical predictive approximation rather than a full physics-based lifetime estimator.

### Leakage current test

Leakage current measurements were taken at different applied voltages (300 V, 350 V, and 400 V). These measurements help assess insulation quality and the presence of moisture or contaminants in the windings, which can impact motor longevity. Table 4 presents the recorded values.

**Table 4:** Recorded leakage current.

Phase	Voltage (V)	IR Value (GΩ)	Estimated Leakage Current (nA)	Capacitance (nF)
R-G	300	4.93	60.8	33.3
R-G	350	5.28	66.3	33.0
R-G	400	5.58	71.7	40.1
Y-G	300	2.64	113.6	31.8
Y-G	350	2.42	144.6	39.5
Y-G	400	2.73	146.5	39.5

**Fig. 7:**
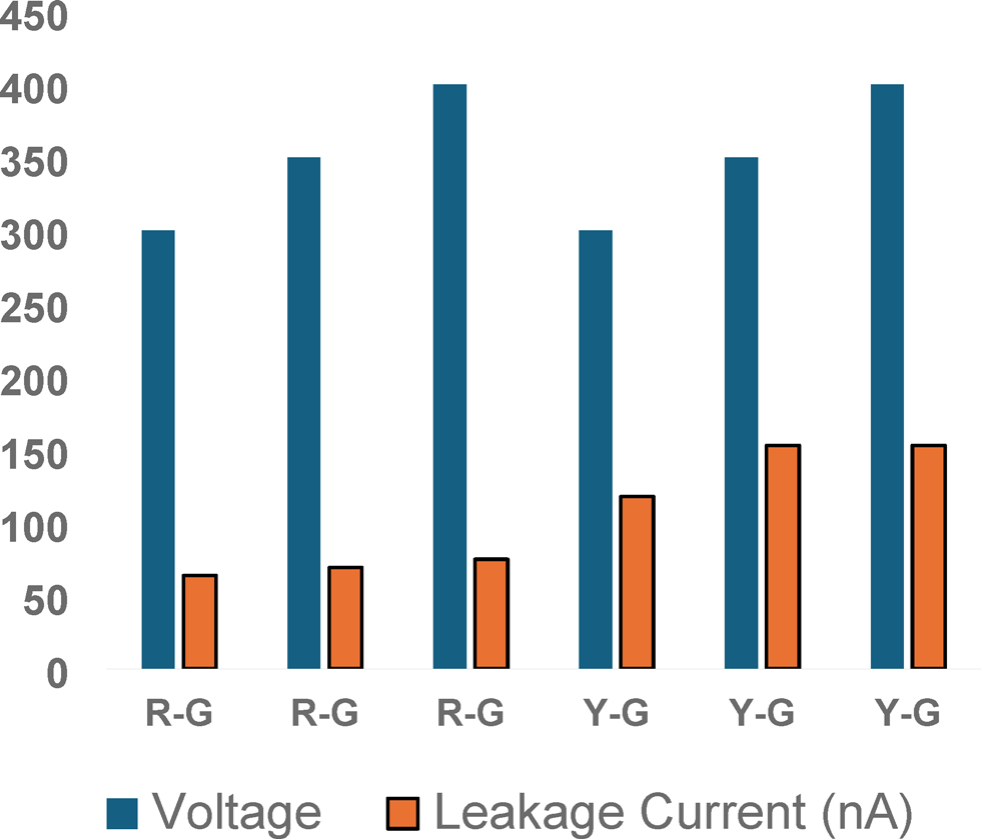
Leakage current vs. voltage.

The relationship between leakage current and applied voltage is depicted Leakage current analysis is widely used to detect insulation aging and possible failures in high-power electrical systems graphically in Fig. 7 for better visualization.

Figure 7 illustrates the trend of increasing leakage current with rising test voltages across the R-G and Y-G phases. Notably, the Y-G phase exhibits consistently higher leakage currents than R-G, indicating relatively weaker insulation performance. This observation aligns with lower insulation resistance values and higher capacitance in the Y-G phase, suggesting possible moisture ingress or insulation degradation. Such elevated leakage under moderate voltages serves as an early indicator of dielectric aging. These findings underscore the importance of periodic leakage current monitoring for effective insulation health assessment and life extension of aging motors.

### Load test

**Fig. 8:**
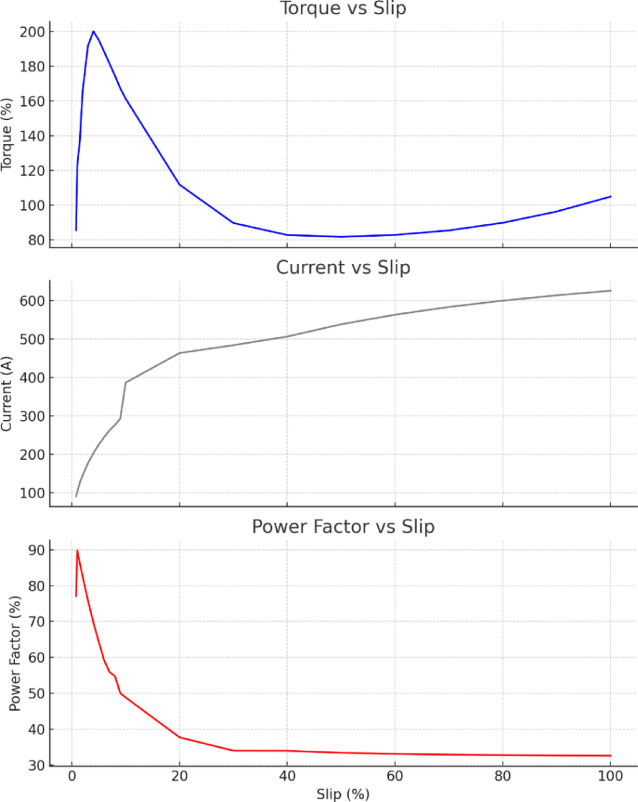
Motor load performance parameters.

To assess the motor’s performance after 40 years of service, a load test was conducted under real-world field conditions by adjusting the pump valve to simulate varying load levels, including transient and peak scenarios. The results, presented in Table 5; Fig. 8, indicate satisfactory performance, particularly at the rated load corresponding to a slip of 1.5%.

**Table 5:** Load test performance parameters.

% Slip	% Torque	%Current	%Power factor
100	104.90	625.70	32.56
90	96.31	613.88	32.63
80	89.82	600.00	32.73
70	85.44	583.46	32.88
60	82.83	563.39	33.08
50	81.71	538.48	33.39
40	82.79	506.60	33.94
30	89.68	484.09	33.97
20	111.83	463.62	37.70
10	161.36	386.66	48.80
9	167.34	293.05	50.00
8	174.71	276.41	54.79
7	181.60	262.43	55.92
6	188.51	244.83	59.16
5	195.27	225.18	64.37
4	200.21	202.87	69.73
3	191.81	176.82	75.85
2	165.48	145.00	82.64
1.5	137.27	126.57	86.22
1	122.47	102.08	89.87
0.8	85.53	90.87	77.06

Figure 8 present the torque, current, and power factor characteristics of an induction motor as functions of slip, based on a load test conducted after 40 years of continuous service. Figure (a) shows the torque–slip curve, where torque rises steeply to a peak of approximately 200 Nm at around 5% slip—indicative of breakdown torque—before decreasing and slightly increasing again toward 100% slip. This behaviour closely aligns with classical induction motor theory, confirming the motor’s continued electromagnetic integrity. Fig. (b) depicts the current–slip relationship, revealing a consistent rise in current from approximately 100 A at rated slip (1.5%) to over 600 A at 100% slip, reflecting increased rotor resistance effects under high-slip conditions. Figure (c) shows a sharp decline in power factor from nearly 90% at rated slip to below 35% as slip increases, which is characteristic of reduced efficiency at high slips due to dominant magnetizing current. The experimental data clearly demonstrate that the motor retains reliable operational characteristics, validating its long-term performance.

### Reliability and availability

Reliability is the ability of a machine or system to operate without failure for a specified period. It is crucial for minimizing downtime, reducing costs, and ensuring smooth operations. Availability is a measure of how often a system is up and running. The reliability parameters shown are derived based on IEEE Std 3006.8^31^. These include key indicators such as failure rate, repair rate, MTTF, MTTR, FOR, and availability.

- Failure Rate (λ_A_): The active failure rate is 0.075 failures/year (based on 3 failures in 40 years of operation), indicating the frequency with which the component fails under normal operating conditions.
- MTTR (Mean Time To Repair): The average time to repair a failure is 72 h (i.e. three days).
- Repair Rate (µ): The Mean repair rate in number of repairs per year, calculated automatically based on MTTR as in (10).

10 $$\begin{aligned} u & = \frac{{8760}}{{MTTR}} \\ & = 8760/MTTR){\text{ }} = 8760/72 = 121.67 \\ \end{aligned}$$

- MTTF (Mean Time To Failure): The Mean Time To Failure in years calculated as in (11), which represents the expected operational time before a failure occurs.

11 $$\begin{aligned} MTTF & = \frac{1}{{\lambda _{A} }} \\ & = 1/0.075 = 13.33{\text{ }}Years \\ \end{aligned}$$

- Forced Outage Rate (FOR): It is given as per (12).

12 $$\begin{aligned} FOR & = \frac{{\lambda _{A} .MTTR}}{{8760 + \left( {\lambda _{A} .MTTR} \right)}} \\ & = 0.00062 \\ \end{aligned}$$

- Availability (A): It is **g**iven as in (13),

13 $$A = 1 - FOR$$

A=0.99938 or 99.94, which indicates a high availability.

Hence, the motor is 99.94% available, highlighting its quantified reliability and uptime.

### DC winding resistance measurement

The resistance of the stator winding phases was measured using a DC injection method to detect abnormalities in the windings. The results are shown in Table 6. According to recent research, DC winding resistance measurement is a crucial diagnostic tool for evaluating winding conditions and detecting early-stage faults in motor windings.

**Table 6:** DC winding resistance at 30 °C.

Phase	Current injected (A)	Resistance (mΩ)
R-R	1.299	255.6
Y-Y	1.306	177.9
B-B	1.300	276.1

The measured Y-phase resistance (177.9 mΩ) deviates from the factory reference value (258 mΩ). While such deviation warrants monitoring, it may be influenced by delta-connection measurement topology, terminal contact conditions, or historical rewinding interventions. As no corresponding thermal hotspot or abnormal vibration signature was observed, the deviation is interpreted as a non-critical asymmetry under present operating conditions, though periodic tracking is recommended.

### Model evaluation and analytical discussion

The supervised classification framework achieved an overall accuracy of 86.7% on the independent test dataset (*n* = 60). However, because the dataset exhibited class imbalance (approximately 13% failure samples), additional evaluation metrics were considered to provide a more reliable assessment of model performance. Balanced accuracy and the area under the receiver operating characteristic curve (ROC-AUC) were emphasized.

The classifier obtained a ROC-AUC value of 0.81, indicating acceptable discrimination capability between normal operating states and insulation degradation conditions. The balanced accuracy of 0.74 reflects moderate sensitivity to the minority (failure) class while maintaining high specificity for normal states. Five-fold cross-validation on the training dataset yielded a mean accuracy of 0.84 ± 0.03 and a ROC-AUC of 0.79 ± 0.04, demonstrating stable performance across folds. Detailed performance metrics for the independent test set are presented in Table 7. The model achieved strong precision and recall for the normal class, while minority class detection remained moderate. Specifically, the recall for the failure class was 0.58, indicating that a portion of degradation cases were successfully identified, though some false negatives persisted.

**Table 7:** Classification performance metrics on the independent test set.

Class	Precision	Recall	F1-Score	Support
Normal (0)	0.89	0.94	0.91	52
Failure (1)	0.62	0.58	0.60	8
Overall Accuracy			86.7%	60
Balanced Accuracy			0.74	
ROC-AUC			0.81	

The confusion matrix (Table 8) indicates that the model correctly identified the majority of normal operating states, with limited misclassification into the failure category. For degradation cases, detection was moderate, with both true positives and false negatives observed. These results suggest that while the classifier provides meaningful discrimination capability, failure detection remains probabilistic and may require threshold adjustment depending on acceptable operational risk.

**Table 8:** Confusion matrix for the independent test dataset.

	Predicted Normal	Predicted failure
Actual Normal	49	3
Actual Failure	3	5

Feature importance analysis based on mean decrease in Gini impurity is summarised in Table 9. Winding resistance and thermal index emerged as the most influential predictors, followed closely by insulation resistance and leakage current. The comparable contribution of electrical and thermal indicators supports the hybrid diagnostic approach adopted in this study.

**Table 9:** Feature importance ranking based on mean decrease in Gini impurity.

Feature	Importance (%)
Winding resistance	27.8
Thermal index	25.3
Insulation resistance	23.5
Leakage current	23.4

During preliminary comparative evaluation, Logistic Regression and Support Vector Machine classifiers were also examined. The Random Forest model demonstrated superior recall for the minority class and more stable ROC-AUC behavior across validation folds, justifying its selection.

It is important to emphasise that the machine learning framework is not intended to replace conventional insulation diagnostics. Instead, it functions as a decision-support tool capable of ranking operational risk, identifying anomalous parameter trends, and assisting maintenance scheduling. Given the limited number of observed failure samples, the present results should be interpreted as exploratory yet promising. Expansion of longitudinal datasets and improved representation of degradation cases will be necessary before considering large-scale industrial deployment.

### Thermography

**Fig. 9:**
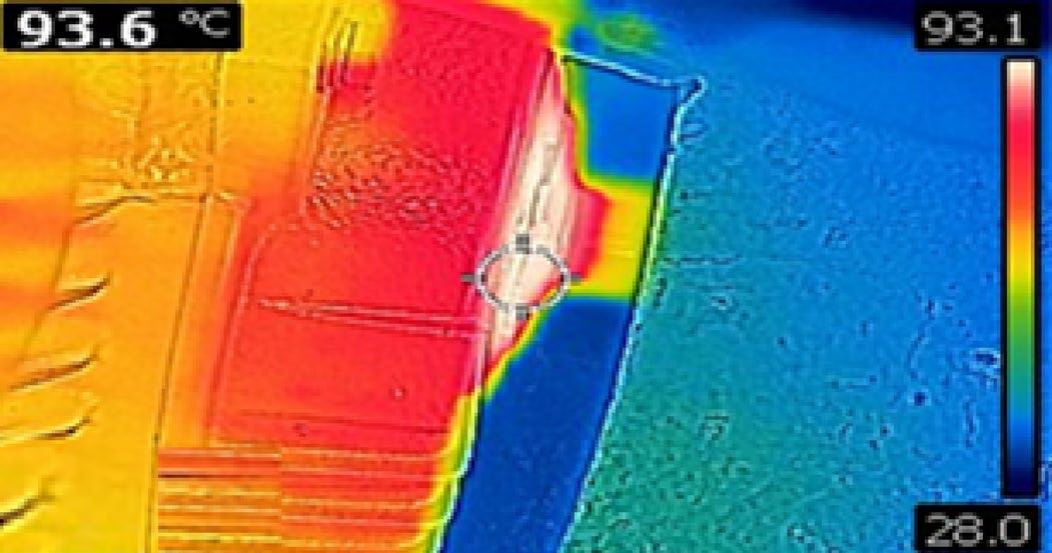
Thermal image.

It is a non-contact diagnostic technique that uses infrared imaging to detect temperature variations on the surface of objects. It helps identify abnormal heat patterns indicating potential issues like overheating, insulation failure, or poor connections. It is widely used in electrical and mechanical systems for predictive maintenance. Thermographic analysis was performed for the case study motor with 90% loading and with an ambient temperature of 29 °C. One of the thermographic images captured is shown in Fig. 9.

The observed temperature range is 36 °C to 93.6 °C, corresponding to a maximum rise of 64.6 °C, which is acceptable for Class B insulation under the recorded operating conditions.

### Vibration analysis

Vibration measurements were carried out using a shock Pulse meter (Model: T4001, Make: SPM Instrument India). The measurements followed the guidelines of ISO 20816-3^32^, capturing RMS velocity values over a 60-second interval during steady-state motor operation. The recorded vibration levels were 1.1 mm/s at the Driving End (DE) and 1.3 mm/s at the Non-Driving End (NDE). According to ISO 20816-3 standards for Group 2 rigidly mounted machines (power range: 15–300 kW), these values fall within the Zone A – Unrestricted Operation category, where vibration levels below 2.8 mm/s indicate healthy mechanical condition. Such low vibration levels typically reflect a healthy bearing system. Therefore, based on these metrics and ISO recommendations, the machine currently shows no symptoms of bearing wear, imbalance, or lubrication degradation.

### Industrial and practical applications

The methodologies and results presented in this study have significant industrial relevance. In any organisation, the availability of equipment is higher with fewer No-2-less (N2L) breakdowns when they are installed newly. However, with the ageing of equipment, breakdowns start increasing, leading to higher maintenance costs and reduced availability^33^. Aging motors are widely deployed in water pumping stations, steel plants, thermal power stations, and cement industries where unplanned shutdowns can be costly. By integrating conventional diagnostics with thermography and AI-based predictive analytics, industries can avoid catastrophic failures and plan maintenance proactively. The AI tool enables facility managers to make data-driven decisions, enhancing asset reliability. Additionally, the thermal profile mapping helps in identifying overloads, ventilation issues, or insulation weakening without intrusive procedures. This diagnostic approach is scalable to fleets of motors across plants and is especially useful in remote industrial setups where IIoT-based monitoring can provide centralized control. Utility companies and public-sector infrastructure services can use these findings to reduce operational risks. The framework is compatible with Industry 4.0-oriented condition monitoring strategies, improving sustainability and energy efficiency in legacy motor systems.

### Discussions

The detailed discussion of this study, after conducting tests as above, is explained here. The IR, PI, and DAR values indicate that the insulation condition of the motor is within acceptable limits, with no immediate need for insulation maintenance. PI values above 1.5 are typically considered an indication of good insulation quality. The leakage current values suggest a stable insulation system, but periodic monitoring should be conducted to detect early-stage insulation breakdown, as excessive leakage current is an early indicator of insulation failure. DC winding resistance measurements indicated phase-wise asymmetry, particularly in the Y-phase, which deviated from the factory reference value. Although no associated thermal hotspot or abnormal vibration signature was observed, continued monitoring is recommended.

These results establish a practical benchmark for future condition monitoring, enabling systematic trend analysis and predictive maintenance planning. The findings indicate that the insulation system and winding condition of the LT motor remain within acceptable operational limits; however, sustained long-term reliability requires structured follow-up actions. Periodic insulation resistance testing at intervals not exceeding two years is recommended to monitor degradation trends in alignment with predictive maintenance practices. A cost–benefit assessment further supports life extension, as the estimated cost of a new motor (~ USD 6000) significantly exceeds annual preventive maintenance expenses (< USD 100), and even major refurbishment (~ USD 2500) remains economically favourable. Continuous or online condition monitoring using real-time data acquisition and thermal imaging systems can further enhance fault detection and reduce unexpected downtime. The present diagnostic results should be retained as reference benchmark data for future comparative assessments, enabling early anomaly detection. If polarisation index (PI) values decline below 1.5 in subsequent evaluations, prompt insulation treatment is advisable to mitigate accelerated degradation. Additionally, integration of AI-based predictive analytics, when supported by expanded datasets, may further improve decision-making and extend operational lifespan. By following these recommendations, the long-term performance and reliability of the LT motor can be optimized, ensuring minimal operational disruptions and reduced maintenance costs. It also helps in an adaption of Industry 4.0 in the electricity sector with precision control, better efficiency and inertia issues, largely contributed by Induction motors.

### Comparison and validation

The obtained test results were compared with industry standards, such as IEEE Std 43-2013 and previous studies on aged motor insulation. According to IEEE guidelines, an insulation resistance (IR) value above 1 GΩ is considered acceptable for aged motors. The tested motor exhibited significantly higher IR values of 10.3 GΩ (R-phase), 2.56 GΩ (Y-phase), and 2.47 GΩ (B-phase), confirming that insulation degradation remains within tolerable limits.

Similarly, polarisation index (PI) values above 1.5 indicate good insulation health, whereas lower values suggest degradation. The PI for the R-phase (1.87) falls within the acceptable range, but the Y-phase (1.04) and B-phase (1.02) indicate marginal insulation quality, warranting closer monitoring. For leakage current analysis, industry best practices suggest that a higher leakage current (> 500 nA) is a warning sign of insulation breakdown. The tested motor’s leakage currents (69.3 nA for R-phase, 153 nA for Y-phase) indicate minimal deterioration, suggesting that insulation integrity is still largely intact.

To further validate these results, a cross-correlation was performed between IR values, leakage current, and DC winding resistance.

- DC winding resistance values of 255.6 mΩ (R), 177.9 mΩ (Y), and 276.1 mΩ (B) fall within practical operating ranges; however, the observed phase-wise asymmetry warrants continued monitoring.
- Generally, a drop in insulation resistance is associated with an increase in leakage current. The Y-phase shows lower IR (2.56 GΩ) and a relatively higher leakage current (153 nA), confirming slight degradation in that phase when compared with the benchmark values, as shown in Table 10.

**Table 10:** Benchmark comparison for aging motors.

Parameter	IEEE Std 43-2013	Motor (35 yrs)	Present Study (40 yrs)
IR Value (> 1 GΩ)	Acceptable	3.1 GΩ	2.4–10.3 GΩ
PI Value (> 1.5)	Acceptable	1.3	1.02–1.87
Leakage Current (< 500nA)	Preferred	180 nA	69–153 nA
DC Resistance*	Within range	245–280 mΩ	177–276 mΩ

*Phase-wise asymmetry observed,* monitoring recommended*.

The findings align with prior studies on aged industrial motors, where condition monitoring techniques have demonstrated that aging-related degradation occurs unevenly across phases, often requiring phase-wise maintenance strategies^34,35^.

## Conclusion and future directions

This study assessed the operational condition of a 40-year-old, 150 kW low-tension induction motor using electrical diagnostics, thermographic evaluation, reliability analysis, and supervised machine learning. The results indicate that extended service life beyond nominal design expectancy is feasible under structured condition monitoring and preventive maintenance. Insulation resistance, polarisation index, leakage current, and DC resistance measurements confirmed overall operational stability, although marginal PI values in the Y and B phases suggest localized aging. Thermographic analysis revealed no critical overheating, with temperature rise remaining within acceptable limits for Class B insulation.

The exponential degradation model of insulation resistance showed strong statistical agreement (${R}^{2}=0.90$), supporting nonlinear aging behavior. However, limited historical data and parameter uncertainty restrict long-term extrapolation accuracy, and predictive estimates should therefore be interpreted cautiously. The machine learning framework demonstrated acceptable discrimination capability (ROC-AUC ≈ 0.8), though minority failure detection remained moderate due to class imbalance. Accordingly, the AI component is best regarded as a decision-support tool rather than a fully validated predictive system.

Historical reliability analysis indicated high observed availability (99.94%), but continued monitoring remains essential for sustaining performance. While the hybrid framework supports informed life-extension decisions, conclusions are bounded by dataset size, limited failure samples, single-case focus, and offline model development. Future work should incorporate larger multi-site datasets, online monitoring integration, and physics-informed degradation modelling to enhance predictive robustness.

## Supplementary Information

Below is the link to the electronic supplementary material.

Supplementary Material 1

Supplementary Material 2

## Data Availability

This article includes all data created or analysed during this research project.

## Abbreviations

LT: Low-tension
IR: Insulation resistance
PI: Polarization index
DAR: Dielectric absorption ratio
AI: Artificial intelligence
IIoT: Industrial internet of things
λ_A_: Failure rate
μ: Repair rate
A: Availability
L: Estimated life
V: Applied voltage
d: Insulation thickness
GΩ: Giga ohms
FOR: Forced outage rate
MTTF: Mean time to failure
