# Supplementary material for: Assessment of a 40-year-old induction motor using hybrid diagnostic and AI-based predictive techniques
Source: Sci Rep. 2026 Mar 17;16:13739. doi: 10.1038/s41598-026-44319-5 (PMC13125488; doi:10.1038/s41598-026-44319-5)
Supplement: Supplementary file 1 — Supplementary Material 1 [file 41598_2026_44319_MOESM1_ESM.docx]

**Highlights**

**•** Assesses a 40-year-old, 150 kW LT motor's viability beyond its 25-year standard lifespan.

• Integrates AI-driven predictive maintenance with thermographic and IR/PI/DAR analysis, reducing unexpected failures by 40%.

• Establishes a diagnostic benchmark for aging motor reliability, aligning with Industry 4.0 and IIoT-based monitoring practices.
