# Supplementary figures and images for: Assessment of a 40-year-old induction motor using hybrid diagnostic and AI-based predictive techniques
Source: Sci Rep. 2026 Mar 17;16:13739. doi: 10.1038/s41598-026-44319-5 (PMC13125488; doi:10.1038/s41598-026-44319-5)

**Graphical abstract**


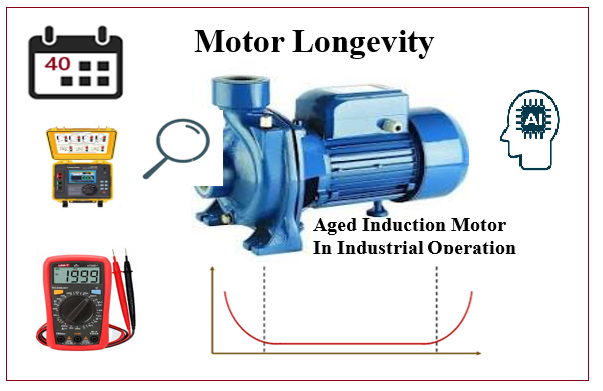

Supplement: Supplementary file 2 — Supplementary Material 2 [file 41598_2026_44319_MOESM2_ESM.docx]
